# Comprehensive overview of the role of PBX1 in mammalian kidneys

**DOI:** 10.3389/fmolb.2023.1106370

**Published:** 2023-03-17

**Authors:** Fei Zou, Mingsheng Liu, Yutong Sui, Jinyu Liu

**Affiliations:** ^1^ Department of Toxicology, School of Public Health, Jilin University, Changchun, China; ^2^ Department of Pediatrics, First Hospital of Jilin University, Jilin University, Changchun, China

**Keywords:** PBX1, kidney, renal carcinoma, development, blood vessels

## Abstract

Pre-B-cell leukemia homeobox transcription factor 1 (PBX1) is a member of the TALE (three-amino acid loop extension) family and functions as a homeodomain transcription factor (TF). When dimerized with other TALE proteins, it can act as a pioneer factor and provide regulatory sequences *via* interaction with partners. In vertebrates, PBX1 is expressed during the blastula stage, and its germline variations in humans are interrelated with syndromic anomalies of the kidney, which plays an important role in hematopoiesis and immunity among vertebrates. Herein, we summarize the existing data on PBX1 functions and the impact of PBX1 on renal tumors, PBX1-deficient animal models, and blood vessels in mammalian kidneys. The data indicated that the interaction of PBX1 with different partners such as the *HOX* genes is responsible for abnormal proliferation and variation of the embryonic mesenchyme, while truncating variants were shown to cause milder phenotypes (mostly cryptorchidism and deafness). Although such interactions have been identified to be the cause of many defects in mammals, some phenotypic variations are yet to be understood. Thus, further research on the TALE family is required.

## 1 Introduction

PBX1 was originally identified as part of a fusion protein caused by chromosomal translocation t (1; 19) in pre-B cell acute lymphoblastic leukemia. It was later renamed to *PBX1* to distinguish it from the prolactin gene *PRL*-*1* (Kamps et al., 1991). *PBX1* encodes the PBX1 protein, and along with PBX2–4 belongs to a family of highly conserved homeodomain proteins (Monica et al., 1991; Wagner et al., 2001). It includes a homeodomain that usually presents three α-helices and a motif that enables PBX proteins to interact with DNA (Qian et al., 1989; Kissinger et al., 1990; Gehring et al., 1994). As part of the TALE family, PBX1 plays critical roles in embryogenesis, organogenesis, development, and differentiation (Wright et al., 1989). Our previous studies have reported that PBX1 facilitates hair follicle-derived mesenchymal stem cell DNA damage repair and attenuates senescence and programmed cell death (Jiang et al., 2019; Liu et al., 2019; Wang et al., 2020; Wang et al., 2021; Liu et al., 2022a; Wang et al., 2022a). Loss-of-function research on mice indicated that PBX1 is necessary for embryo growth and survival, since PBX1-knockout embryos reached embryonic lethality at about half-a-month post-pregnancy (Kim et al., 2002; Schnabel et al., 2003a). The mouse embryos lacking PBX1, which is also a key regulator of renal morphogenesis, were also shown to have mesenchymal dysfunction, which further leads to kidney developmental damage (Schnabel et al., 2003a). Moreover, it has been reported that *PBX1* promotes tumor progression as an oncogene (Zhou et al., 2020; Lee et al., 2021; Liu et al., 2022b).

A large number of studies have been reported on human diseases caused by mutant *PBX1* pathogenic genes such as asplenia (Arts et al., 2020), pulmonary hypoplasia (Alankarage et al., 2020; Arts et al., 2020), microsplenia (Arts et al., 2020), heart malformation or congenital heart disease (Le Tanno et al., 2017; Alankarage et al., 2020; Arts et al., 2020), bilateral diaphragmatic eventration (Arts et al., 2020), dysmorphic features (Le Tanno et al., 2017; Riedhammer et al., 2017; Alankarage et al., 2020; Arts et al., 2020), bone malformation (Riedhammer et al., 2017; Alankarage et al., 2020), dysmorphic features (Le Tanno et al., 2017; Riedhammer et al., 2017; Alankarage et al., 2020; Arts et al., 2020), sex reversal (Arts et al., 2020), cryptorchidism (Le Tanno et al., 2017; Riedhammer et al., 2017), neurological malformation (Le Tanno et al., 2017), global developmental delay (Riedhammer et al., 2017), growth retardation (Riedhammer et al., 2017; Alankarage et al., 2020), inborn defects of the kidney and urinary tract (Le Tanno et al., 2017; Riedhammer et al., 2017; Arts et al., 2020), poor prognosis (Magnani et al., 2011), and intellectual disability (Riedhammer et al., 2017; Alankarage et al., 2020). These prior studies demonstrate the importance of PBX1 in the development and the function of major tissues and organs. In this meta-analysis, we provide a comprehensive overview of the role of PBX1 in mammalian kidneys.

## 2 Results

### 2.1 PBX1 in renal carcinoma

The aberrant PBX1 expression is associated with poor prognoses (Magnani et al., 2015), tumorigeneses (Park et al., 2008), resistance to cancer therapy (Jung et al., 2016), and poor response to induction therapy (Veselska et al., 2019). Initial PBX1 is associated with cancers of the hematopoietic system (Shimabe et al., 2009), which are followed by solid tumors such as neuroblastoma (Veselska et al., 2019), renal carcinoma (Wei et al., 2018; Wang et al., 2022b), gastric cancers (He et al., 2017), and esophageal cancers (Zhu et al., 2017).

The “Oncomine” database shows that abnormal PBX1 overexpression exists in numerous cancer categories, including renal clear cell carcinomas (ccRCCs), accounting for 70% of renal tumors (Zhang et al., 2016; Hsieh et al., 2017; Wei et al., 2018). Furthermore, the expression of PBX1 was dynamically upregulated in four renal carcinoma cell lines (OS-RC-2, KAKi-2, 786-O, and 769-P) and the HK-2 proximal renal tubule cell line (Wei et al., 2018). There was significantly lower survival among patients with high PBX1 expression in the nucleus and cytoplasm of ccRCC cells than in those with low PBX1 expression. This may be related to the fact that PBX1 promotes the phosphorylation of STAT3Tyr705 to activate the JAK2/STAT3 signaling pathway in ccRCC. The decrease in STAT3Tyr705 phosphorylation after PBX1 knockout in ccRCC cells may also influence cancer cell growth and play an important role in renal tumor development (Wei et al., 2018).

It has been found that more than 80% of ccRCC tumors exhibit epigenetic changes or possess genetic changes in the von Hippel–Lindau (VHL) gene (Nickerson et al., 2008; Moore et al., 2011; Sato et al., 2013). Interestingly, in ccRCC patients with VHL mutations, PBX1 mRNA expression levels and PBX1 transcriptional scores were also associated with their clinical outcomes. Higher PBX1 transcription scores were associated with better total survival, whereas the decreased expression of PBX1 was associated with poor VHL mutation prognosis in ccRCC, which may be related to the molecular heterogeneity of the VHL-mutant ccRCC subgroup (Wang et al., 2022b). Another study by Margon et al. showed that HOX along with PBX could affect apoptosis of the renal cancer lines CaKi-2 and 769-P (Shears et al., 2008). The results of these studies indicate that PBX1 may be a novel prognostic factor for renal tumors and has potential applications in treating human renal tumors.

### 2.2 PBX1 in renal development

PBX1 encodes a TALE homeodomain transcription factor that regulates basic developmental processes in many different tissues, including the kidneys and spleen. It exists in structures derived from the intermediate mesoderm throughout all stages of urogenital system ontogeny and is expressed in the renal interstitium, nucleus medulla, and interstitial area and then into the posterior renal interstitium (Schnabel et al., 2001; Schnabel et al., 2003a; Schnabel et al., 2003b). Studies have found high expression of PBX1 in stromal cells and low expression of PBX1 in nephron progenitor cells (Schnabel et al., 2001; Schnabel et al., 2003a; Hurtado et al., 2015; Le Tanno et al., 2017), of which the latter is a direct target of Six2/Brg1 playing an important role in adjusting the fine balance between cell cycle progression and cell cycle maintenance (Li et al., 2021).

Since *HOX* genes are modified by PBX1 to perform certain functions and can play an important role in mammalian kidney development (Davis et al., 1995; Mann and Chan, 1996; Patterson et al., 2001; Wellik et al., 2002), it can be speculated that PBX1 and PBX regulatory protein families form nuclear complexes to improve the DNA-binding specificity of HOX proteins and regulate transcription during embryonic development (Slavotinek et al., 2017). In PBX1-deficient mice, the interference with this transcription factor is related to nephrogenesis (Schnabel et al., 2003a; Hurtado et al., 2015). However, that variation is restrained in PBX1 mutants with expanded areas of mesenchymal condensates, which contain a preponderance of cycling cells.

While PBX1-deficient kidneys lead to nephrogenesis, they have sustained proliferation of the mesenchyme without subsequent differentiation. In mild PBX1 mutant cases, ureteric buds have been shown to invade and branch, resulting in delayed renal vesicle formation, thicker cap condensate, and expanded c-ret expression (Schnabel et al., 2003a). Moreover, among most mutants, bilateral kidney formation is abnormal and manifests as delayed nephrogenesis and ureteral branch defects (Schnabel et al., 2003a).

The expression patterns described previously suggest that PBX1 may play an important role in fetal renal development. Using RT-qPCR, Le Tanno found that PBX1 was highly expressed in kidneys during the fetal period and was also relatively highly expressed in the brain during embryonic development. However, its expression was downregulated in adult kidneys (Le Tanno et al., 2017). The GUDMAP database displays that PBX1 is just highly expressed in not only renal tumor cells (for example, A-498, ACHN cell lines, and so on) but also in renal epithelial cells (for example, HK-2, RPTEC/TERT1 cell lines and so on).

PBX1 is crucial for interstitial–epithelial signal transduction, an important regulator of interstitial function in renal morphogenesis (Le Tanno et al., 2017), and critical for renal morphogenesis and development. For example, patients with pathogenic PBX1 mutations/microdeletions exhibited multifarious alloplasia (Slavotinek et al., 2017), and PBX1-deficient mice had delayed nephrogenesis and ureteral branching defects (Schnabel et al., 2003a). Congenital anomalies of the kidney and urinary tract (CAKUT) are diseases with various phenotypes presenting with congenital anatomical abnormalities of the urinary system. These include abnormal development of the kidney, ureter, bladder, and posterior urethra and constitute the main cause of chronic kidney disease in children caused by genovariation (Capone et al., 2017). PBX1 has been identified as a monogenic cause of CAKUT in mammals, with Le Tanno *via* microarray analysis previously reporting pathogenic variation in microdeletion-related genes as a cause of renal agenesis, with PBX1 as the smallest common region (Le Tanno et al., 2017). So far, more than 32 different pathogenic PBX1 variants have been reported. Renal agenesis, hyperechogenicity, pelvicalyceal dilation, bilateral nephroureters, ectopic kidneys, horseshoe-shaped kidneys, bilateral vesicoureteral reflux, and small urethral valves are common PBX1 mutant renal phenotypes, though renal agenesis is rarer. In the study by Petzold et al. (2022), chronic kidney disease stage 3 was observed at the time of the onset except in two adult patients with bilateral renal hypoplasia. Most patients were younger than 5 years of age at the time of their first diagnosis, and their renal functions declined variably. PBX1 mosaicism presents with a mild course and sporadicalness in CAKUT cases.

### 2.3 PBX1 in kidney blood vessels

PBX1 plays a pivotal role in promoting self-renewal and coordinating the extent of proliferation with the terminal differentiation of progenitor cells (Selleri et al., 2001; Ficara et al., 2008). Selleri et al. (2001) speculated that the loss of its function would lead to abnormal development of the cardiovascular system, and PBX1-null embryos in mice have displayed abnormal great artery morphogenesis due to failed establishment of initial complement of the branchial arch arteries (Chang et al., 2008; Stankunas et al., 2008). Hurtado et al. (2015) showed that PBX1 in renal vascular mural progenitor cells directly represses PDGFRB, which is a master initiator of vascular mural cell–blood vessel association in mice. Premature differentiation of vascular cells is associated with non-productive angiogenesis, abnormal renal artery branching, significant disturbance of the renal artery tree structure, and renal dysfunction. Moreover, ablation of PBX1 considerably remedies vascular patterning defects.

As one of the most unique transcription factors, PBX1 was found to have enriched expression or activity in glomerular capillaries, and its induction coincided with the ontology of the glomerulus (Barry et al., 2019). PBX1 may be recruited by renal epithelial cells to prune the gene expression that dominates vascular zonation from the embryonic period to the adult stage and appropriately adjust the specialized functions of the glomeruli (Barry et al., 2019).

## 3 Conclusion

**FIGURE 1:**
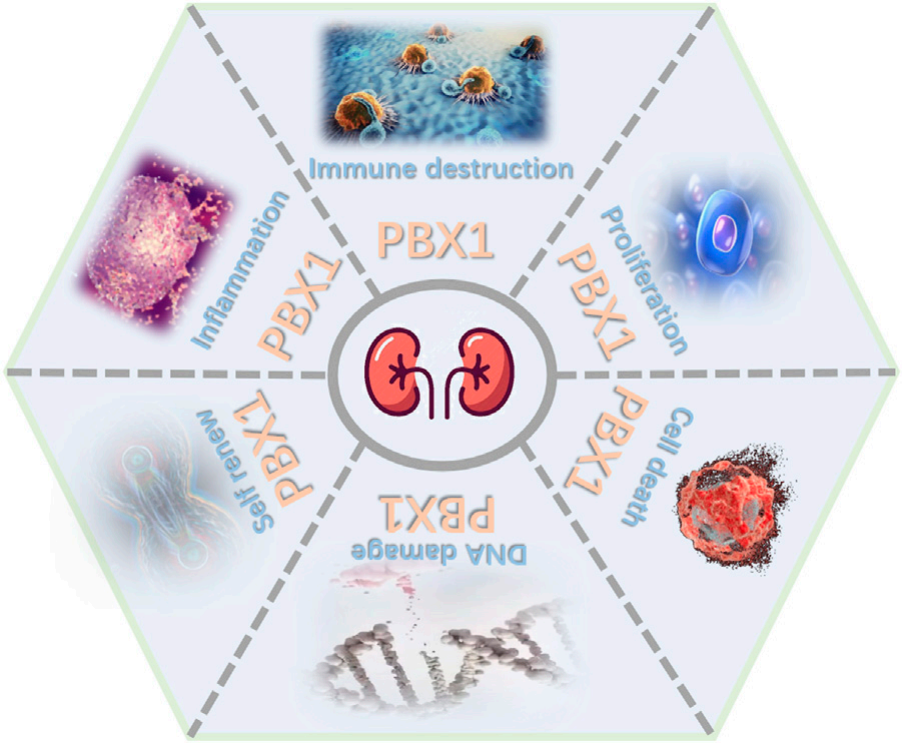
Major roles of PBX1. PBX1 contributes to kidney development, tumor progression, and blood vessels by interfering in proliferation, cell death, self-renewal, DNA damage, inflammation, and immune destruction (figure was modified from Servier Medical Art (http://smart.servier.com/).

The role of PBX1 is complex, with diverse contributions. As transcription factors, PBX1 and its partners together influence the occurrence and development of various diseases. As mentioned previously, PBX1 functions in mammalian kidneys include promoting tumor progression, regulating basic developmental processes, and adjusting glomeruli vascularization. Most of the time, PBX1 as an oncogene promotes the proliferation of tumor cells, induces angiogenesis, and participates in the occurrence and development of tumors. Mutation and disorder of PBX1 will cause serious and pleiotropic consequences such as CAKUT (Figure 1). Therefore, further research should help us understand the specific involvement of PBX1 in each type of kidney disease and to consider the renal pathophysiological changes caused by an altered PBX1 expression or pathogenic mutations. The therapeutic effects of some small-molecular drugs targeting specific diseases have been proved effective, but the relevant research on PBX1 as a molecular drug still need to be carried out. Importantly, years of research on this protein have set the foundations for currently expanding biotech and industrial activity devoted to turning such knowledge into treatments for patients.
